# Pocket Endocrinology

**Published:** 1988-08

**Authors:** Ian Bailey


### POCKET CONSULTANT ENDOCRINOLOGY

**Martin Hartog, D. M., F.R.C.P.**
**Blackwell Scientific Publications, 1987**
**Pages 179**

I have carried this useful book in my pocket and used it with benefit in clinical meetings. It is intended for hospital doctors as a guide to practical management of people with diabetes and endocrine disorders. It describes each gland, the normal function and its variants, the symptoms and signs, the tests which might be used to establish the diagnosis and treatment. There is a sensible approach to the use of tests and there is helpful practical advice on the management of diabetes and its complications and on measures to control hyperlipidaemias. The book's usefulness outside the hospital is limited except for the management of the common disorders of diabetes and upset thyroid function.

The book is well produced and could usefully be used by students and by postgraduates reading for the Membership.

IAN BAILEY

